# A Missed Opportunity: Prioritizing the Development of a Healthy Market Ecosystem for Equitable Menstrual Health Within the International Conference on Population and Development Programme of Action

**DOI:** 10.9745/GHSP-D-24-00125

**Published:** 2025-08-14

**Authors:** Sarah Webb, Tanya Mahajan

**Affiliations:** a Reproductive Health Supplies Coalition, Washington, DC, USA.; b Menstrual Health Action for Impact, New Delhi, India.; c The Pad Project, New Delhi, India.

## Abstract

Prioritizing the development of a healthy market ecosystem for equitable menstrual health within the International Conference on Population and Development Programme of Action can ensure that individuals have access to destigmatized, unbiased, and complete information, products, and services they need to manage their menstrual health.

## Key Messages

- Menstrual health is inextricably linked to outcomes outlined in the International Conference on Population and Development (ICPD) Programme of Action to accelerate reproductive health outcomes and strive for a world of gender equity.
- Without elevating access to menstrual health and establishing an enabling environment to create access to menstrual health information, products, services, and facilities, the bold vision of ICPD will not be possible.
- A pathway for addressing these outcomes is facilitating healthy market structures for destigmatized, unbiased access to menstrual health information, products, services, and facilities and cross-sectoral impact on areas such as family planning, water and sanitation, and education.
- To ensure sustained access and cross-sectoral impact, investments to strengthen menstrual health markets should prioritize innovation and choice, explore integrated delivery of products and services (beyond just menstrual products), and address structural market barriers across the value chain, with leadership from key stakeholders in the Global South.

## MENSTRUAL HEALTH: A MISSING LINK TO ACCELERATE THE INTERNATIONAL CONFERENCE ON POPULATION AND DEVELOPMENT PROGRAMME OF ACTION

In 1994, the International Conference on Population and Development (ICPD) placed sexual and reproductive health and rights (SRHR) at the forefront of the global agenda. From Cairo, 179 governments pledged support for the bold Programme of Action, calling upon the global community to accelerate efforts to support family planning (FP) and maternal health and to recognize the innate linkages between women’s reproductive health (RH) and economic empowerment and participation.^1^ In the years since, the ICPD Programme of Action has seen various iterations, adaptations, and new calls to action as new evidence emerges, demographic trends shift, efforts stall, and new sociopolitical trends take center stage. Despite new commitments made under its many iterations—the original 1994 Programme of Action, 20th Anniversary Edition, and the Nairobi Summit commitments from 2019— an important commitment that is intricately linked with outcomes related to SRHR, education, and women’s economic participation and gender equity has always been absent: menstrual health (Box 1).^2^^–^^5^

BOX 1 Definition of Menstrual Health

“Menstrual health is a state of complete physical, mental, and social well-being and not merely the absence of disease or infirmity, in relation to the menstrual cycle.”^5^ This definition of menstrual health recognizes the importance of a holistic, comprehensive understanding of menstrual health that encompasses:

- Accurate information about menstrual health
- Access to menstrual health products and services that prioritizes individual preferences, comfort, and privacy
- Menstrual discomforts and disorders and relevant diagnostics and treatment, including for pain management
- A supportive, respectful environment free from stigma
- Freedom from menstrual-related exclusion, discrimination, coercion, and violence

Yet, menstrual health, in its comprehensive definition for policy and practice, plays a critical role in delivering on the bold ICPD Programme of Action.^5^ Sidelined by pervasive stigma and taboo and coupled with a lack of data, menstrual health was long siloed away from broader sexual and reproductive health (SRH) and gender equity. Only in the last 15 years has menstrual health started to receive global attention as a catalyst for RH, education, economic, and gender equity outcomes, following incredible efforts by passionate advocates across the globe and catalytic funding and research.^5^ During the 2019 Nairobi Summit on ICPD25, several global advocacy organizations, including The Case for Her and WASH United, pushed for menstrual health’s inclusion in the renewed ICPD commitments, and there were a few isolated examples of country governments adopting menstrual health commitments (e.g., South Africa).^6^^,^^7^ Now, it’s time for menstrual health to have a place in the official ICPD Programme of Action as we look ahead to the next 30 years. Activists, advocates, and leaders from the Global South have been instrumental in the progress to date and will continue to be important in leading the charge.^8^

While quantitative data around menstrual health access and linkages to gender equity, economic advancement, education, and health outcomes is still limited—in large part due to the lack of investment in data generation—there is a growing body of qualitative evidence from the Global South that documents the status of poor menstrual health and alludes to these linkages.^9^^,^^10^ There are also efforts to generate good quality quantitative data to understand these linkages further.^11^ First and foremost, menstrual health access is an issue of gender equity, dignity, and human rights for those who menstruate. Throughout this article, we use the term “those who menstruate” instead of women and girls to emphasize the important intersection of gender and recognize the complexities of gender identity with menstruation.

Menstrual health access is an issue of gender equity, dignity, and human rights for those who menstruate.

Approximately 26% of the global population are women of reproductive age. Every day, an estimated 300 million of these women are menstruating.^12^ Lack of information, products, services, and facilities for the management of menstruation and related issues contribute to reduced participation at school and work, poor RH outcomes, and increased risk of gender-based violence (GBV).

Globally, 100 million girls of high school age are out of school.^13^ In 2018, global women’s labor force participation at 48.5% was still 26.5 percentage points below their male counterparts.^14^ The proportion of adolescent girls and young women who are not in education, employment, or training is twice as high for female youth compared to males in low- and middle-income countries (LMICs).^15^ Access to safe, affordable, and quality menstrual products when needed, water and sanitation facilities, knowledge of pain management strategies, and a supportive ecosystem are needed to ensure engagement at school and workplaces. These gaps lead to distraction, disengagement, and absenteeism from school and work for those who menstruate.^16^^,^^17^ In low-income contexts, where education and economic participation for girls and women is not a priority, this sets the stage for school dropouts and skewed labor force participation.^18^

Menstruation also marks the beginning of the reproductive cycle, inextricably linking menstrual health to broader SRH outcomes. Menstrual health conversations can improve health-seeking behaviors and symptomatic identification of reproductive tract and sexually transmitted infections, menstrual disorders (endometriosis, fibroids, and cysts), identification pipeline for cervical cancer, and appropriate and continued use of cycle-based and hormonal contraceptive methods. Menstrual health awareness can also be used to strengthen self-care for maternal health, including managing postnatal bleeding, expected changes in the menstrual cycle, and symptomatic identification of infections and managing perimenopause and menopause, including preventing and managing cardiovascular, musculoskeletal, mental, and noncommunicable disease health issues.^19^^,^^20^

Thirty-five percent of women globally experience GBV, and 200 million women have experienced female genital mutilation.^21^ Stigmatization of menstruation is associated with and cumulatively leads to the lack of psychosocial agency over hygiene and reproductive practices of those who menstruate and also has far-reaching impacts on factors like age of marriage and first childbirth.^22^ Cumulatively, this lack of agency, starting with prepubertal gender norms, increases the vulnerability of those who menstruate over the reproductive life course. The stigma and silence around menstruation lend themselves to poor body literacy and loss of psychosocial agency, which, in contexts where harmful practices like female genital mutilation and other forms of GBV are prevalent, can make those who menstruate more vulnerable to these practices.^22^^–^^24^ Addressing gaps in access to information, products, services, and facilities for menstrual health is a critical step toward addressing these cross-sectoral outcomes.

While those working in the field of menstrual health are starting to take note of these linkages, practical approaches for achieving these outcomes are still extremely limited.^19^^,^^20^^,^^25^ An underexplored pathway for influencing these intersections is the development of a healthy and well-functioning market for menstrual health products and services. Such a market keeps those who menstruate as the focus and aims to offer choice and agency in information, products, and services through the democracy of the market.

## A PATHWAY FORWARD FOR MENSTRUAL HEALTH THROUGH A HEALTHY MARKET ECOSYSTEM

A healthy market for menstrual health products, services, and facilities can ensure that those who menstruate have (1) access to unbiased and comprehensive information about them; (2) proximal availability to them as part of a supportive ecosystem; and (3) psychosocial agency to negotiate for and access them when needed, across their reproductive life course for the management of menstrual bleeding, pain, disorders, and other issues (Box 2).

BOX 2 Menstrual Health Products, Services, and Facilities Defined

Menstrual health products, services, and facilities include but are not limited to:

- A range of single and multiple use and externally and internally used bleeding management products
- Diagnostic, counseling, and treatment-related health services
- Pharmaceutical solutions for management of menstrual pain, concerns, menopause, premenstrual syndrome, and premenstrual dysphoric disorder
- The full range of family planning methods
- Water and sanitation facilities for menstrual health and hygiene management, including for product disposal

Accelerating access to menstrual health for influencing cross-sectoral outcomes will require dedicated investment and coordinated efforts across sectors to address systemic barriers to the development of these markets. The emphasis on leveraging market-based approaches to realize the bold ambition of the ICPD Programme of Action and expand the quality and range of RH services is also well-established. In fact, the ICPD Programme of Action highlights the important role of market-based approaches in ensuring the realization of the reproductive rights and RH agenda, highlighting the important opportunity to engage the private sector, develop cost-recovery strategies, and leverage the market to improve accessibility.^2^ To develop a healthy market ecosystem for menstrual health, the following different investment areas should be explored.

Accelerating access to menstrual health for influencing cross-sectoral outcomes will require dedicated investment and coordinated efforts across sectors to address systemic barriers to the development of these markets.

### Investment in Menstrual Health Should Prioritize Product Innovation and Choice

Historically, menstrual health markets have been focused on the provision of single-use menstrual pads. However, with new investments in market development, there is an opportunity to prioritize product choice and innovation around new products.^26^ Just like not all contraceptive users have the same preferences around contraceptive products, those who menstruate have diverse needs and preferences. Product choice not only plays a critical role in the uptake and use of menstrual products but also plays an important role in ensuring that all those needs across contexts and the reproductive life course are met.^27^ In doing so, choice has the potential to expand the market by including new consumers. For example, a large proportion of those who menstruate in LMICs use cloth-based solutions. This, combined with recent studies on the acceptability of multiple-use solutions, offers the opportunity to expand the market to offer washable pads, menstrual underwear, and menstrual cups. Product innovation and choice will also pave a pathway toward minimizing the economic and environmental cost of menstrual products.^28^^,^^29^

### Investment in Menstrual Health Markets Need to Go Beyond Menstrual Products

Health outcomes related to menstruation require the provision of products, services, and facilities beyond just products for managing bleeding. Solutions for the management of menstrual bleeding and pain, menstrual concerns, reproductive tract and sexually transmitted infections, and the uptake and use of contraceptive methods can be offered through the same channels by exploring innovative access models. While these products and services may be available, approaches for integrated provision have not yet been explored extensively. Investments in menstrual health markets need to prioritize this to not only realize the associated SRH, maternal, and other health outcomes but also use economies of scope at the last mile to reduce the cost of provision of integrated services. For example, integrating FP counseling to include menstrual health awareness or contraception access models to include menstrual products can help in achieving both outcomes while minimizing investments.^25^ These market-based approaches can create new opportunities for integration, leveraging market dynamics to bundle additional products and services related to FP and RH. With growing attention on opportunities to improve private-sector engagement and provision of FP and RH commodities and services—including within the ICPD Programme of Action—this opportunity to integrate menstrual health and streamline cost-effective investment will be particularly important.^30^ Innovations in solutions are also needed to expand the basket of services available to consumers. Menstrual health awareness, including body literacy and other aspects of menstrual health, is also critical for addressing demand-side barriers and creating a supportive ecosystem to achieve these outcomes. Innovations in supportive facilities in household and institutional settings (e.g., menstrual-friendly water, sanitation, and hygiene facilities) are also necessary for the hygienic use of a basket of products.

### Investment in Menstrual Health Should Address Structural Market Barriers

The market for menstrual products (for the management of bleeding) has long been a consumer-driven market, where products are predominantly accessed by those who menstruate purchasing them rather than through free distribution, donation mechanisms, or bulk procurement by governments.^31^ This is likely, in part, because menstrual health has not been integrated into broader health programs and universal health coverage mechanisms, but it also presents a unique market opportunity that will result in better long-term sustainability. Currently, many of those who menstruate (particularly in LMICs) face affordability and availability barriers in purchasing an adequate supply of menstrual products. However, with 1.72 billion people who menstruate across LMICs and only 59% of those using purpose-made products for all their product needs, there is significant potential for market development.^32^ Efforts to address structural barriers (e.g., misaligned tax structures, disaggregated supply chains for raw materials and finished products, lack of standards for products, and better understanding of the willingness and, importantly, ability of those who menstruate to pay) are necessary for long-term downward trends in consumer prices. This will also ensure that when free distribution of products is necessary (e.g., during humanitarian crises or among particularly vulnerable populations), resources are spent more efficiently—both through the reduction of product cost and by shifting donor or government expenditures to those most in need.

### Investment in Menstrual Health Should Create Opportunities for New and Emerging Manufacturers in the Global South

Menstrual product supply chains are fraught with dependencies on imports from the Global North and South East Asia due to a lack of local technological and manufacturing capacity. As investment into menstrual health markets accelerates, it will be critical to ensure that these investments provide the impetus for regionalized research and development and manufacturing capacity, coupled with enacting policies incentivizing them for a range of menstrual health products and services at various levels of scale and scope. As markets become more efficient and upstream cost drivers are mitigated, new manufacturers will be better positioned to enter and compete in local markets.^31^

### Investment in More and Better Data Is Critical

Primary data around menstrual health demand and supply and the impact of menstrual health on economic empowerment, RH outcomes, and education remains limited. While there are tremendous anecdotal stories and lived experiences that paint a clear picture of the impact of menstrual health, better data are necessary to develop strong business cases, incentivize investment, and demonstrate the return on investment for menstrual health. Yet, data generation, collation, and analysis are expensive and require tremendous advocacy.

## CALLING ON THE GLOBAL COMMUNITY TO PRIORITIZE MENSTRUAL HEALTH

Now that the global community has celebrated 30 years of ICPD, it is time to ensure that menstrual health is not only included in the ICPD agenda but also prioritized and funded. To achieve the ICPD Programme of Action, menstrual health is a necessary ingredient and a prerequisite for achieving gender equity, educational advancement, economic empowerment, SRHR and eliminating GBV, discrimination, and bias. By investing in the development of healthy markets for menstrual health, we will not only ensure that those who menstruate worldwide have access to the information, products, and services that they need to manage their menstruation but also ensure that that access remains equitable and sustainable. Let us not miss another opportunity to elevate menstrual health in the global agenda.

## Notes

**Peer Reviewed**
